# KEGG orthology-based machine learning reveals functional determinants of antimicrobial resistance in *Acinetobacter baumannii*

**DOI:** 10.1128/spectrum.02592-25

**Published:** 2026-05-07

**Authors:** Zhihang Zheng, Bei Jiang, Abebe Mekuria Shenkutie, Jingyuan Bian, Yuyao Yan, Ruizhen Pi, Qing Xiong, Polly Hang-Mei Leung

**Affiliations:** 1 Department of Health Technology and Informatics, The Hong Kong Polytechnic University, Kowloon, Hong Kong SAR, China; Shenzhen University School of Medicine, Shenzhen, China

**Keywords:** antimicrobial resistance, KEGG orthology, machine learning, *Acinetobacter baumannii*, functional genomics

## Abstract

Antimicrobial resistance represents a critical global health threat, with *Acinetobacter baumannii* classified as a priority pathogen due to its extensive multidrug resistance. Currently, antimicrobial susceptibility testing relies on phenotypic characterization, which provides limited mechanistic insights into resistance mechanisms and significantly delays appropriate therapy. While machine learning approaches have shown promise for rapid resistance prediction, existing computational methods rely predominantly on gene presence-absence matrices that inadequately capture functional capacity. To address this limitation, we developed a functional genomics framework using KEGG orthology annotations to predict antimicrobial resistance phenotypes directly from whole-genome sequencing data. We systematically evaluated six machine learning algorithms on 1,088 *A. baumannii* isolates across 10 antimicrobial agents, with subsequent validation in an independent cohort of 508 isolates. KEGG orthology-based representations consistently outperformed traditional gene presence-absence encoding across all algorithms. XGBoost achieved superior performance (94.44% weighted accuracy) and was therefore selected for interpretability analysis. Identified determinants included known carbapenemases and, crucially, K18974 (*sul1*), a ubiquitous marker of integron-mediated propagation. Additionally, K21801 (*iaaH*) was identified as a key driver of non-conventional resistance via indole-mediated signaling. Subsequent multidrug resistance analysis identified shared determinants—overlapping KEGG orthologs across antimicrobial classes—that generate predictable cross-resistance patterns. These computationally derived molecular networks closely mirrored the observed clinical co-resistance phenotypes. Overall, this interpretable machine learning framework advances mechanistic understanding of resistance evolution and establishes a promising computational foundation. Future prospective validation is required to translate these findings into clinical utility.

**IMPORTANCE:**

*Acinetobacter baumannii* represents one of the most urgent antimicrobial resistance threats globally, and traditional diagnostic methods can take up to 48 hours to determine appropriate treatment options. This delay contributes to poor patient outcomes and drives further resistance development through inappropriate antibiotic use. This study addresses a critical gap by developing a machine learning framework that can rapidly predict antimicrobial resistance patterns directly from whole-genome sequencing data. Unlike previous approaches that rely on simple gene presence-absence data, our method uses functional genomic annotations to capture the complex biological processes underlying resistance. The framework not only achieves superior prediction accuracy but also provides interpretable insights into resistance mechanisms, identifying both known resistance genes and previously unrecognized metabolic pathways involved in antimicrobial resistance. The biological insights gained can open new avenues for developing targeted therapeutic interventions.

## INTRODUCTION

Antimicrobial resistance (AMR) represents one of the most significant public health challenges of the 21st century, with projections indicating it will soon surpass cancer as a leading threat to global health (1, 2). The rapid emergence and spread of multidrug-resistant (MDR) bacterial pathogens have created an urgent need for innovative approaches to detect, predict, and combat resistance (3). Among the most concerning MDR pathogens is *Acinetobacter baumannii*, a gram-negative bacterium classified by the World Health Organization as a critical priority pathogen due to its remarkable ability to acquire resistance to multiple antibiotic classes (4).

However, current clinical methods for antimicrobial susceptibility testing (AST) require up to 48 hours to generate reliable results, significantly delaying the implementation of appropriate antibiotic therapies (5). This delay not only impacts patient outcomes but also contributes to the inappropriate use of broad-spectrum antibiotics, further driving the development of resistance.

Recent advances in high-throughput sequencing technologies have enabled comprehensive analysis of bacterial genomes, creating opportunities for the application of machine learning approaches to predict AMR phenotypes directly from genomic data (6–8). Most previous research relies on the presence-absence matrices of genes and single-nucleotide polymorphisms (SNPs) (9, 10), which have established themselves as the predominant methods for resistance prediction and phylogenetics, respectively. However, both approaches inadequately capture the functional capacity of microbial systems (11–13). While reliable for known resistance mechanisms, they consequently fail to predict resistance arising from novel or uncharacterized elements. This is particularly problematic given that hypothetical proteins, which are abundant in bacterial genomes, may harbor these unidentified resistance determinants.

Kyoto Encyclopedia of Genes and Genomes (KEGG), widely accepted as the most accurate database for functional annotation, is also known for its large pathway information (14). Within the KEGG framework, genes with similar functional roles are clustered into KEGG orthologs (KO). These orthologous sets comprise homologous sequences derived from diverse organisms that share common molecular functions, as determined by the KEGG functional annotation criteria. Therefore, in bacterial analysis, KO’s pathway-centric approach helps predict functions for those hypothetical proteins based on their genomic context. Bacterial isolates, even within the same species, exhibit considerable genetic diversity. KO annotation is therefore crucial, as it captures the functional equivalence of genes despite such sequence variations (15). Additionally, KO annotations have proven valuable in evolutionary studies, as they enable the reconstruction of ancestral gene content and facilitate machine learning-based prediction of gene-gain and gene-loss events across diverse phylogenetic clades (16). Furthermore, unlike purely sequence-based classifications, KO contextualizes genes within biological pathways, providing deeper functional insights (17).

**Fig 1:**
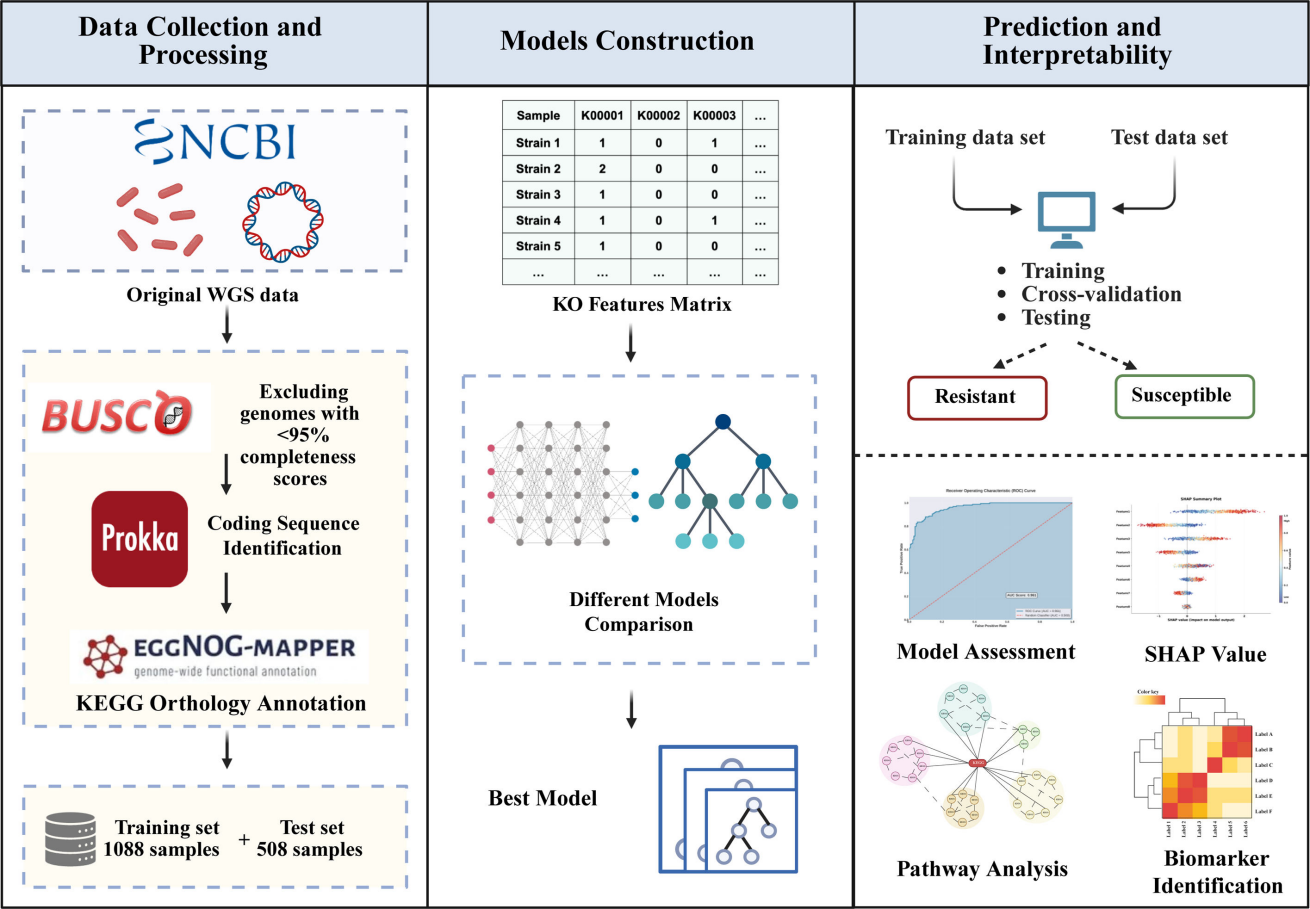
Study overview. A functional genomics-based antimicrobial resistance prediction framework utilizing KEGG orthology (KO) annotations from whole-genome sequencing data to predict resistance phenotypes in *Acinetobacter baumannii*. (Left) WGS data for *A. baumannii* isolates were collected from the NCBI database, with an independent test cohort. Quality control using BUSCO excluded low-completeness genomes. Prokka identified coding sequences, followed by KO annotation via eggNOG-mapper. A KO abundance matrix was constructed based on occurrence frequency across isolates, resulting in training and test data sets with multiple antimicrobial agents. (Middle) Six machine learning algorithms were evaluated. XGBoost demonstrated superior performance and was selected for interpretability analysis. (Right) XGBoost was validated through cross-validation and independent testing for binary resistance classification. Feature importance and SHAP analyses identified key functional determinants, revealing canonical resistance mechanisms and novel metabolic pathways. KEGG pathway enrichment highlighted resistance-related pathways. Multidrug resistance analysis revealed shared genetic architectures, with strong concordance between molecular connectivity and clinical co-resistance phenotypes. Created in https://BioRender.com.

This study comprehensively characterized diverse antibiotic-resistance determinants in multidrug-resistant *A. baumannii* strains and predicted their antimicrobial phenotypes against major antibiotics using KO annotation derived from whole-genome sequencing (WGS). We systematically evaluated six distinct machine learning and deep learning algorithms: Extreme Gradient Boosting (XGBoost) (18), Neural Oblivious Decision Ensembles (NODE) (19), Disjunctive Normal Form Networks (DNF-Net) (20), TabPFN (21)—a transformer-based foundation model for tabular data, Support Vector Machines (SVM) (22), and Random Forest (23). XGBoost demonstrated superior performance and was subsequently validated on an independent test data set to confirm the generalizability and robustness of KO-based resistance prediction. We then employed both XGBoost’s built-in importance and SHAP values to identify key functional determinants of antibiotic resistance. Comprehensive analysis encompassed individual drug-specific features and cross-drug comparisons to elucidate shared resistance mechanisms across multiple antibiotics. This framework (Fig. 1) contributed to the mechanistic understanding of resistance evolution in multidrug-resistant *A. baumannii* and provides a robust foundation for precision antimicrobial therapy strategies in clinical practice.

## MATERIALS AND METHODS

### Data set curation

The training set of antibiotic susceptibility and resistance data for 1,092 *A. baumannii* isolates were collected from NCBI (the list of all isolates and corresponding accession numbers is available in Table S1). Then, in order to validate the performance of the proposed model, we used an independent test set of 521 *A. baumannii* isolates from the literature (24) (accession numbers provided in Table S2). To ensure data quality, we implemented a rigorous preprocessing pipeline for all isolates. For genomic data, we first assessed genome completeness and quality using BUSCO 5.8.2 (Benchmarking Universal Single-Copy Orthologs) (25). Genomes with BUSCO complete scores below 95% were excluded. For AST results, we utilized the phenotypes as provided by NCBI for the training set. For the independent test set, raw MICs (Table S2) were converted to resistant/susceptible categories using EUCAST breakpoints. Following this harmonization, we retained drugs based on two criteria: (i) AST data were available for >40% of the training isolates, and (ii) the minority phenotype (resistant or susceptible) was present in at least 20% of these isolates. In the independent test set, we exclusively included drugs that were retained after applying the aforementioned filtering criteria to the training set to maintain consistency. This filtering approach ensured sufficient representation of drug response variability while maintaining adequate sample size for model training.

**Fig 2:**
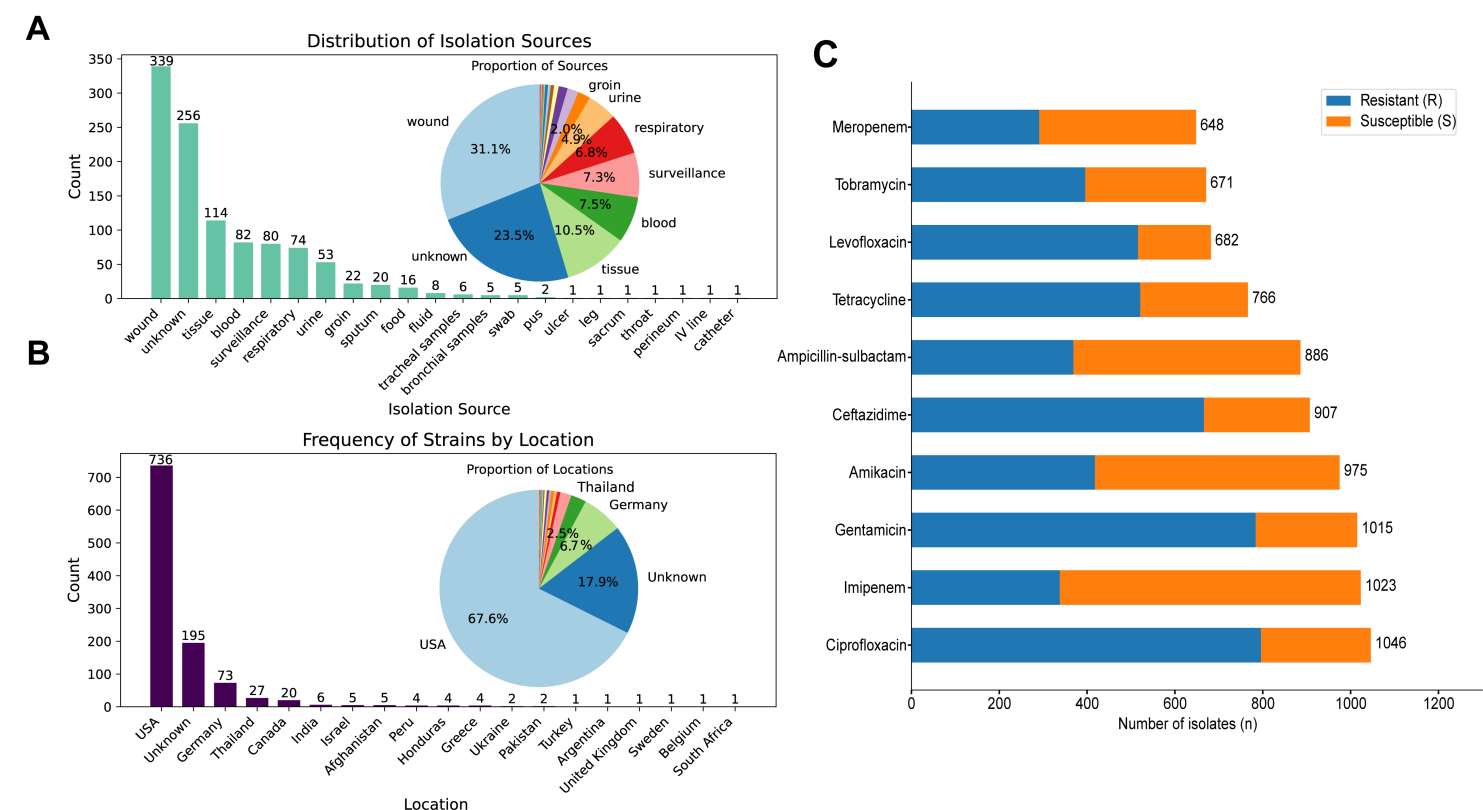
Epidemiological characteristics and antimicrobial resistance patterns of *Acinetobacter baumannii* isolates. (**A**) Distribution of isolation sources (*n* = 1,088). Bar chart shows frequency counts, and pie chart displays proportional distribution. Wound samples constitute the largest proportion (31.1%, *n* = 339). (**B**) Geographic distribution by location (*n* = 1,088). Most isolates originated from the United States (67.6%, *n* = 736), followed by unknown locations (17.9%, *n* = 195), and Germany (6.7%, *n* = 73). (**C**) Antimicrobial resistance profiles showing proportion of resistant (blue) and susceptible (orange) isolates for each antibiotic selected.

After these procedures, a total of 1,088 *A. baumannii* isolates were included in the training set (Table S1), predominantly recovered from wound samples (*n* = 339, 31.1%), followed by unknown sources (*n* = 256, 23.5%) and tissue samples (*n* = 114, 10.5%), with the majority originating from the United States (*n* = 736, 67.6%) and Germany (*n* = 73, 6.7%) (Fig. 2A and B). Phylogenetic analysis revealed distinct clustering patterns with several predominant sequence types, including ST-2, ST-3, and ST-32, which formed distinct clades in the maximum-likelihood tree (Fig. S1). These isolates were tested against 10 antimicrobial agents: amikacin (AMK), gentamicin (GEN), tobramycin (TOB), ciprofloxacin (CIP), levofloxacin (LVX), ceftazidime (CAZ), tetracycline (TET), ampicillin-sulbactam (SAM), imipenem (IPM), and meropenem (MEM). Resistance profiles were determined for each isolate-antibiotic combination (Fig. 2C). Furthermore, these procedures resulted in an independent test set of 508 isolates with five antimicrobial agents included in the training set (Table S2). The independent test set originated exclusively from China (24). This distinct geographic distribution provides a rigorous scenario for external validation.

### Annotation of KEGG orthologs

The genomes were then processed using Prokka 1.11 (26) with default parameters for automated annotation to identify coding sequences (CDS). Subsequently, we employed eggNOG-mapper 2.1.12 (27) to assign KO terms to the identified CDS. Annotations were performed using the DIAMOND search mode against the eggNOG v.5.0 database, with a default e-value threshold of 0.001. Then, we constructed a KO abundance matrix using all identified orthologous groups across isolates, wherein each cell value represents the occurrence frequency of individual KO terms within each respective isolate. For baseline comparison, we generated a gene presence-absence matrix by clustering the Prokka-identified genes using Panaroo v.1.0 (28).

### Phylogenetic analysis

Core genome alignment was generated using Panaroo from the training data set isolates and the reference strain *Acinetobacter baumannii* ATCC 19606 obtained from NCBI. SNPs were extracted using snp-sites v.2.5.1 (29), and maximum likelihood phylogenetic trees were constructed using IQ-TREE v.2.1.3 with automatic model selection and 1,000 ultrafast bootstrap replicates (30). Trees were visualized using the Interactive Tree of Life platform (31), rooted with ATCC 19606, and annotated with sequence types determined by MLST using the abaumannii_2 scheme (32–34).

### Model design

Here, we systematically evaluated six distinct machine learning and deep learning algorithms to identify the optimal approach for KO-based antimicrobial resistance prediction, including gradient boosting, differentiable trees, neural logic networks, transformer-based models, kernel methods, and ensemble techniques.

#### XGBoost

XGBoost implements an optimized gradient boosting framework that sequentially builds decision trees to correct prediction errors from previous iterations. The algorithm incorporates L1 and L2 regularization penalties to prevent overfitting and employs intelligent tree pruning strategies that evaluate split quality using second-order gradients. Its handling of missing values through learned directions and built-in feature importance scoring makes it particularly effective for high-dimensional genomic data sets where feature sparsity is common (35).

#### Random Forest

Random Forest constructs ensembles of decision trees using bootstrap sampling and random feature selection at each split. This dual randomization strategy reduces overfitting while maintaining prediction accuracy through variance reduction across the ensemble (36).

#### NODE

NODE builds ensembles of differentiable oblivious decision trees that enable end-to-end gradient optimization. Unlike standard trees, oblivious decision trees apply the same splitting criterion at every node within a given depth level—essentially creating a structured lookup table rather than a traditional branching pattern. The key innovation lies in replacing discrete decision functions with entmax transformations, which allow both feature selection and threshold comparisons to become differentiable while maintaining sparsity (19).

#### DNF-Net

DNF-Net transforms classical Boolean logic into differentiable neural architectures by implementing soft versions of disjunctive normal formulas. The model replaces hard Boolean operations with continuous approximations, enabling gradient-based learning while maintaining logical structure. Each disjunctive normal neural form block combines fully connected transformations with differentiable conjunctive operations, allowing the network to learn complex logical relationships inherent in genomic resistance patterns (20).

#### TabPFN

TabPFN leverages transformer architecture pre-trained on millions of synthetically generated tabular data sets to perform few-shot learning on new classification tasks. Unlike traditional models requiring data set-specific training, TabPFN approximates Bayesian posterior distributions through in-context learning, processing both features and samples simultaneously via attention mechanisms. This foundation model approach enables rapid adaptation to genomic classification tasks without extensive hyperparameter tuning (21).

#### SVM

SVM identifies optimal decision boundaries by maximizing margins between classes in transformed feature spaces using kernel functions. The algorithm’s ability to operate effectively in high-dimensional spaces through the kernel trick makes it well-suited for genomic data analysis, where the number of features often exceeds sample size (37).

Prior to model training, we applied algorithm-specific preprocessing strategies. For SVM, which relies on distance metrics, features were strictly standardized with Z-score normalization. However, for tree-based ensembles and neural architectures, the KO abundance matrix was utilized as raw frequency counts. This deliberate choice preserves the inherent sparsity of the genomic matrix, where a value of zero carries the absolute biological meaning of gene absence. Furthermore, tree ensembles (XGBoost, Random Forest) are mathematically invariant to feature scaling, and other neural networks incorporated internal Batch Normalization layers to dynamically standardize activations during training, obviating the need for prior input scaling.

All model implementation relied on PyTorch (v.2.2.2), scikit-learn (v.1.2.0), XGBoost (v.2.1.4), and TabPFN (v.2.0.9). Hyperparameters were determined through empirical manual tuning based on preliminary validation performance. Deep learning methods (NODE, DNF-Net) utilized standard robust optimization practices to ensure stable convergence, specifically being optimized using the Adam optimizer (learning rate: 0.001, batch size: 32) for 30 epochs. Tree-based ensembles (Random Forest, XGBoost) were configured with 100 estimators; specifically, XGBoost employed a maximum depth of 5 and a learning rate of 0.1. SVM utilized an RBF kernel with standard data scaling. TabPFN was deployed using its official pre-trained interface with probability balancing enabled.

### Evaluating parameters

To comprehensively assess these models, we applied multiple metrics, including Accuracy (ACC), Precision, Recall, Specificity, F1-score, and Area Under the ROC Curve (AUC), which are calculated based on true negatives (TN), true positives (TP), false negatives (FN), and false positives (FP). ACC represents the proportion of correctly classified instances. Precision evaluates the correctness of positive predictions, and the F1-score provides the harmonic mean of Precision and Recall. Recall (also referred to as Sensitivity) measures the ability to identify positive cases, while Specificity quantifies the correct identification of negative cases. AUC evaluates discrimination capability across thresholds by plotting recall against false positive rate (1—specificity), with values ranging from 0.5 (random guessing) to 1 (perfect classification). To ensure robust model evaluation, we employed fivefold cross-validation. To rigorously address the inherent class imbalance across different antimicrobials, we implemented a Random Oversampling strategy. Crucially, to prevent data leakage and ensure an unbiased evaluation, this oversampling was applied exclusively to the minority class within the training subset of each cross-validation fold. The validation sets and the independent test set were left entirely unaltered to reflect true real-world clinical distributions. All experiments here were conducted in Python 3.8.


(1) $$\begin{array}{c} ACC=\frac{TP\;+\;TN}{TP\;+\;TN\;+\;FP\;+\;FN} \end{array}$$

(2) $$\begin{array}{c} Precision=\frac{TP}{TP\;+\;FP} \end{array}$$

(3) $$\begin{array}{c} Recall=\frac{TP}{TP\;+\;FN} \end{array}$$

(4) $$\begin{array}{c} Specificity=\frac{TN}{TN\;+\;FP} \end{array}$$

(5) $$\begin{array}{c} F1=2\times \frac{Precision\;\times \;Recall}{Precision\;+\;Recall} \end{array}$$

(6) $$\begin{array}{c} AUC=\;\int_{0}^{1}TPR\left(FPR\right)d\left(FPR\right) \end{array}$$

## RESULTS

### Models comparison on training set

**Fig 3:**
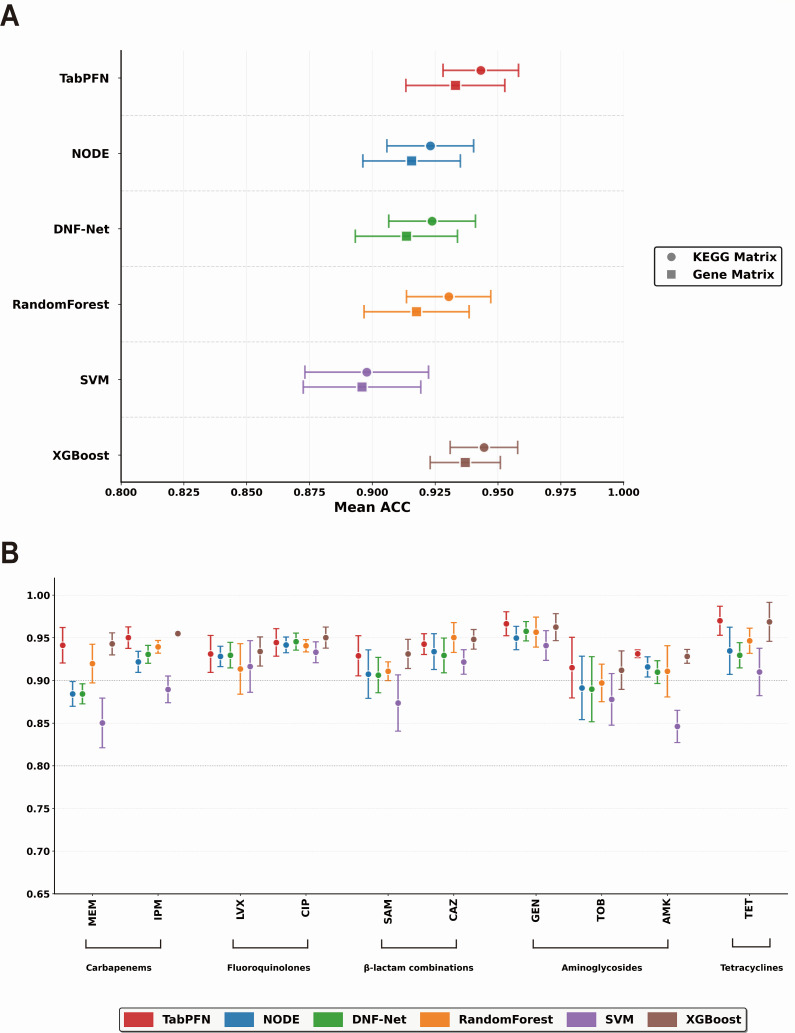
Comparative performance of six models for antibiotic resistance prediction. (**A**) Sample-size-weighted accuracy comparison between KEGG orthology-based (KEGG matrix, circles) and traditional gene presence-absence (gene matrix, squares) representations across six machine learning algorithms. KEGG-based functional annotations consistently outperformed binary gene matrices across all models. XGBoost and TabPFN achieved highest weighted accuracies under functional representation. (**B**) Drug-specific performance evaluation across 10 antimicrobial agents. XGBoost excelled in carbapenem resistance (imipenem and meropenem), while TabPFN dominated for tetracycline and gentamicin. Random Forest demonstrated stable performance across the antibiotic panel. SVM showed limitations, particularly for amikacin and meropenem, compared to ensemble methods.

We first assessed whether KEGG orthology-based representations offer advantages over traditional gene presence-absence encoding. Since our data set contained different numbers of samples for each antibiotic, we calculated overall model performance using sample-size-weighted averages to ensure proportional representation. This weighted analysis revealed that the function-based approach consistently improved prediction accuracy across all six machine learning models (Fig. 3A), with improvements ranging from 0.19% for SVM to 1.02% for DNF-Net. These gains demonstrated that organizing genes by their molecular function captured resistance patterns better than merely cataloging which genes exist. Among all models tested, XGBoost and TabPFN performed best under this functional representation, achieving 94.44% and 94.32% weighted accuracy, respectively.

With KEGG orthology-based representations established as superior, we then examined how different models leveraged these representations across specific antibiotics. As shown in Fig. 3B, XGBoost excelled at carbapenem resistance, achieving 95.50% accuracy for imipenem and 94.29% for meropenem—critical drugs for which prediction errors carry severe clinical consequences. In contrast, TabPFN demonstrated superior performance for aminoglycosides, achieving 96.65% accuracy for gentamicin, 93.13% for amikacin, and 91.50% for tobramycin. For amikacin and meropenem, both TabPFN and XGBoost methods dramatically outperformed SVM by over 8 percentage points, highlighting the limitations of kernel-based approaches for these antibiotics. Furthermore, McNemar’s tests (Table S3) corroborated this dominance, revealing that TabPFN and XGBoost secured the highest frequency of statistically significant victories (*P* < 0.05) against competing architectures across these antibiotics. Notably, Random Forest proved remarkably stable across the antibiotic panel (mean: 93.04%), which underscored that tree ensembles remain competitive for resistance prediction despite lacking the inductive biases of neural architectures. To rigorously validate these findings, we further assessed Precision, Recall, F1-score, and ROC-AUC for each antibiotic (Table S4). These metrics confirmed the superior performance of XGBoost and TabPFN. These complementary strengths suggest that no single model dominates across all resistance phenotypes—a finding with implications for clinical deployment strategies.

### Generalization on test set

**Fig 4:**
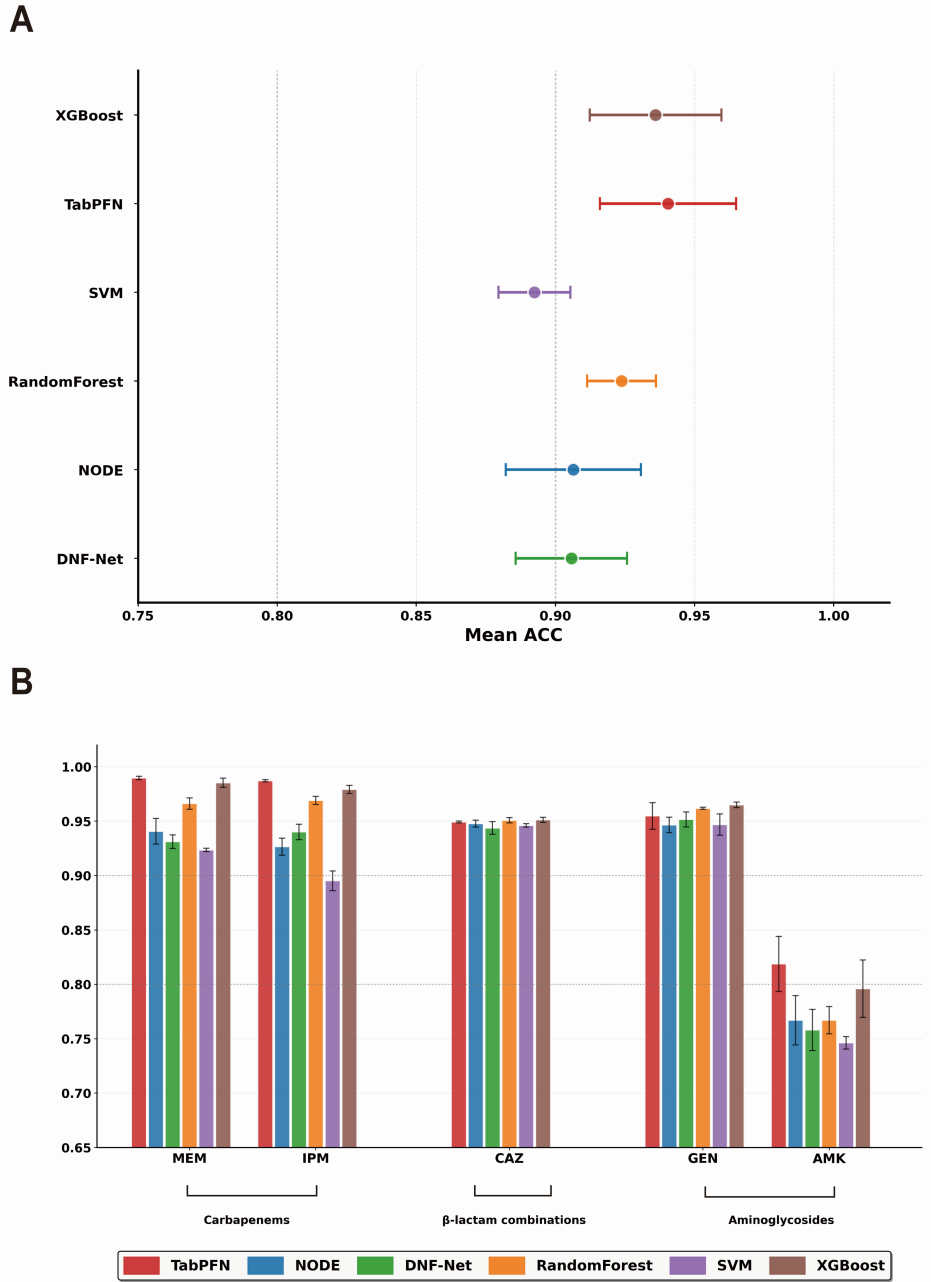
Model generalization performance on independent test set. (**A**) Weighted accuracy comparison across six machine learning algorithms on an independent test cohort of 508 *A. baumannii* isolates. TabPFN achieved highest overall performance, followed by XGBoost and Random Forest, while neural networks (NODE and DNF-Net) showed consistent underperformance. (**B**) Drug-specific performance evaluation across five antibiotics revealed substantial variation in generalization capability. TabPFN maintained superiority on carbapenems (IPM and MEM), while XGBoost dominated for CAZ and GEN. Notably, AMK presented significant generalization challenges across all models, with accuracies dropping substantially compared to cross-validation performance.

We further evaluated model generalization on an independent test cohort of 508 *A. baumannii* isolates across five antibiotics to validate cross-validation findings. The test results largely confirmed our training observations, with TabPFN achieving the highest weighted accuracy (0.940), followed by XGBoost (0.936) and Random Forest (0.924), while neural networks NODE (0.906) and DNF-Net (0.906) showed consistent underperformance (Fig. 4A). This stable ranking across validation and test phases demonstrates that tree-based methods and transformer architectures effectively leverage discrete genomic features, whereas conventional neural networks struggle with KEGG ortholog representations.

Drug-specific analysis revealed substantial performance variations, with some antibiotics showing notable generalization gaps (Fig. 4B). TabPFN maintained its superiority on carbapenems, achieving accuracies of 0.987 (imipenem) and 0.990 (meropenem), while XGBoost continued to dominate ceftazidime (0.951) and gentamicin (0.965). However, amikacin presented an unexpected generalization challenge, with test accuracies dropping to 0.746–0.819 across all models—a significant decline from cross-validation performance where most models achieved competitive results. This amikacin-specific generalization gap suggests either a distribution shift between training and test cohorts or that amikacin resistance mechanisms vary substantially across different *A. baumannii* populations. This divergence was further corroborated by comprehensive performance metrics (Precision, Recall, F1-score, and ROC-AUC) provided in Table S5. While TabPFN and XGBoost maintained exceptional robustness across the other four agents with F1-scores consistently exceeding 0.97, the difficulty in predicting amikacin resistance was evidenced by a marked reduction in discriminatory power, with ROC-AUC values remaining below 0.89 even for the top-performing models. The robust performance of ensemble methods on four of five antibiotics nonetheless reinforces their practical value for clinical deployment.

### Identification of resistance-conferring KEGG orthologs

To identify key KO terms associated with AMR in *A. baumannii*, we employed interpretable machine learning models to quantify feature importance. Given XGBoost’s superior performance in overall resistance prediction tasks during fivefold cross-validation, we selected this algorithm for feature importance analysis. We computed both SHAP values and the model’s built-in feature importance metrics to obtain a comprehensive feature importance profile (Table S6). All calculations were performed on the validation set of each fold. To assess the robustness of these feature attributions, we computed 95% confidence intervals for the SHAP values using a bootstrapping approach on the validation sets (Table S7).

**Fig 5:**
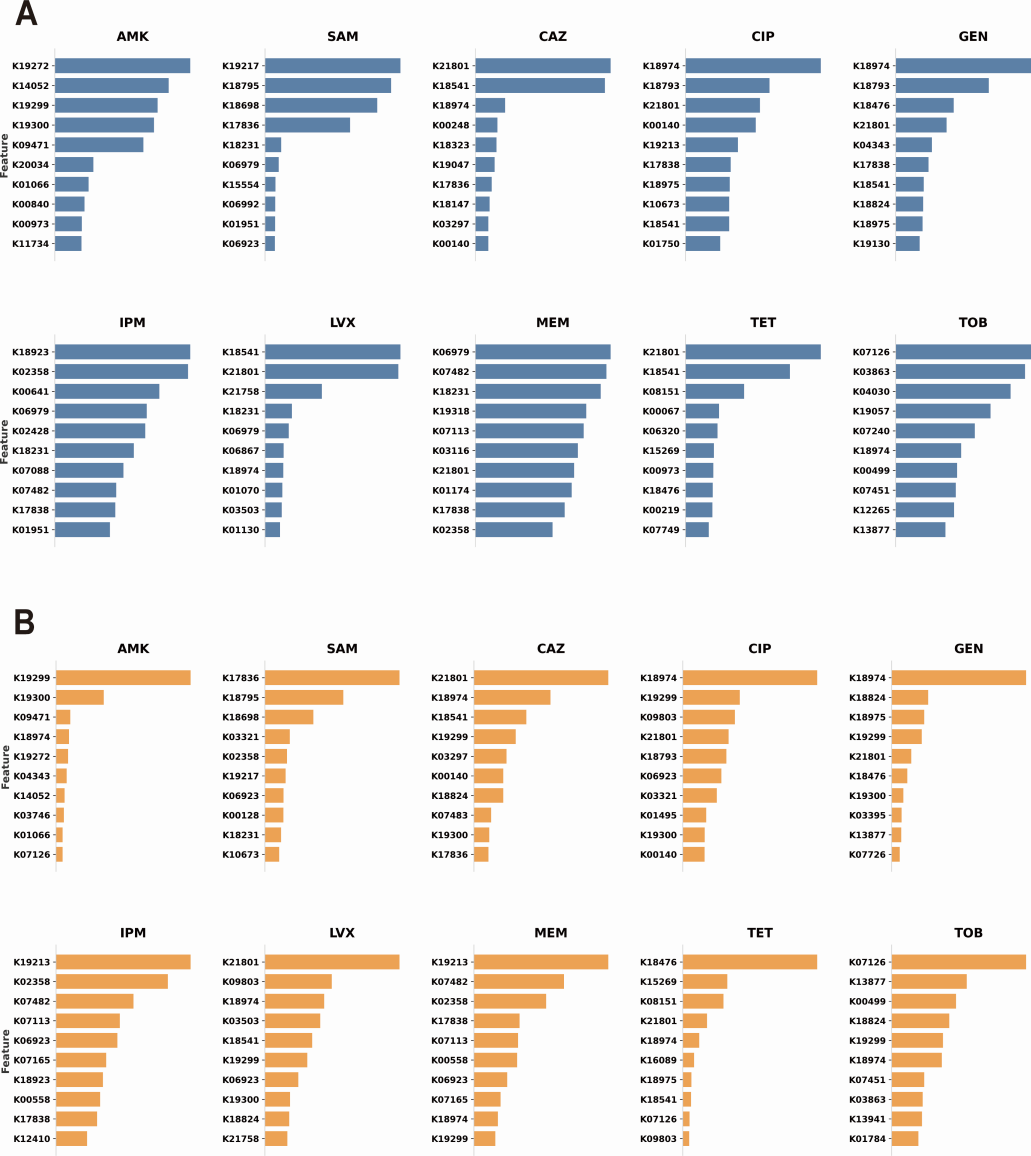
Feature importance analysis of resistance-associated KEGG orthologs. (**A**) Top 10 KO terms for each antimicrobial agent ranked by XGBoost built-in feature importance scores. (**B**) Corresponding rankings based on SHAP values. Both methods identified biologically meaningful resistance determinants, with many high-importance KO terms previously implicated in antimicrobial resistance mechanisms.

Figure 5A presents the top 10 KO terms for each antimicrobial agent based on XGBoost’s built-in feature importance, while Fig. 5B displays the corresponding SHAP-derived rankings. Both analytical approaches revealed drug class-specific patterns in feature importance. For aminoglycosides (e.g., amikacin), the model prioritized enzymatic modification genes, identifying K19272 [*aac(3)-I*] and K19299 [*aph(3*′*)-III*] as top predictors. For fluoroquinolones (LVX and CIP), K21801 (*iaaH*) exhibited higher importance scores. Similarly, for β-lactam combinations (SAM), K17836 (*penP*) was consistently identified as a key determinant. Notably, many of the high-importance KO terms identified by our models have been previously implicated in antimicrobial resistance mechanisms for their respective drugs. For carbapenems (imipenem and meropenem), SHAP analysis consistently identified K19213 (*blaOXA-12*) as the top predictor. For meropenem specifically, this was immediately followed by K07482 (transposase, IS30 family) as the second most important feature. This co-occurrence is biologically revealing: K19213 represents the class D β-lactamases, a family central to *A. baumannii* carbapenem resistance (encompassing intrinsic variants like *blaOXA-51* and acquired types) (38). Crucially, the resistance phenotype of these class D enzymes often requires upstream insertion sequences (such as ISAba1, which belongs to the IS30 family) to provide strong promoters for overexpression (39, 40). Similarly, K18476 (*tetR*) and K08151 (*tetA*) emerged as significant predictors for tetracycline resistance, consistent with their well-established roles in this resistance mechanism. *tetA* encodes an efflux pump that actively exports tetracycline from bacterial cells, while TetR functions as a repressor that regulates tetA expression in response to tetracycline (41). These genes are frequently located on conjugative plasmids and transposons, facilitating horizontal transfer across diverse bacterial genera (42).

Collectively, these interpretability analyses demonstrate that the model not only achieves high predictive performance but also captures biologically meaningful patterns, providing mechanistic insights into the molecular determinants of antimicrobial resistance that align with established microbiological knowledge.

**Fig 6:**
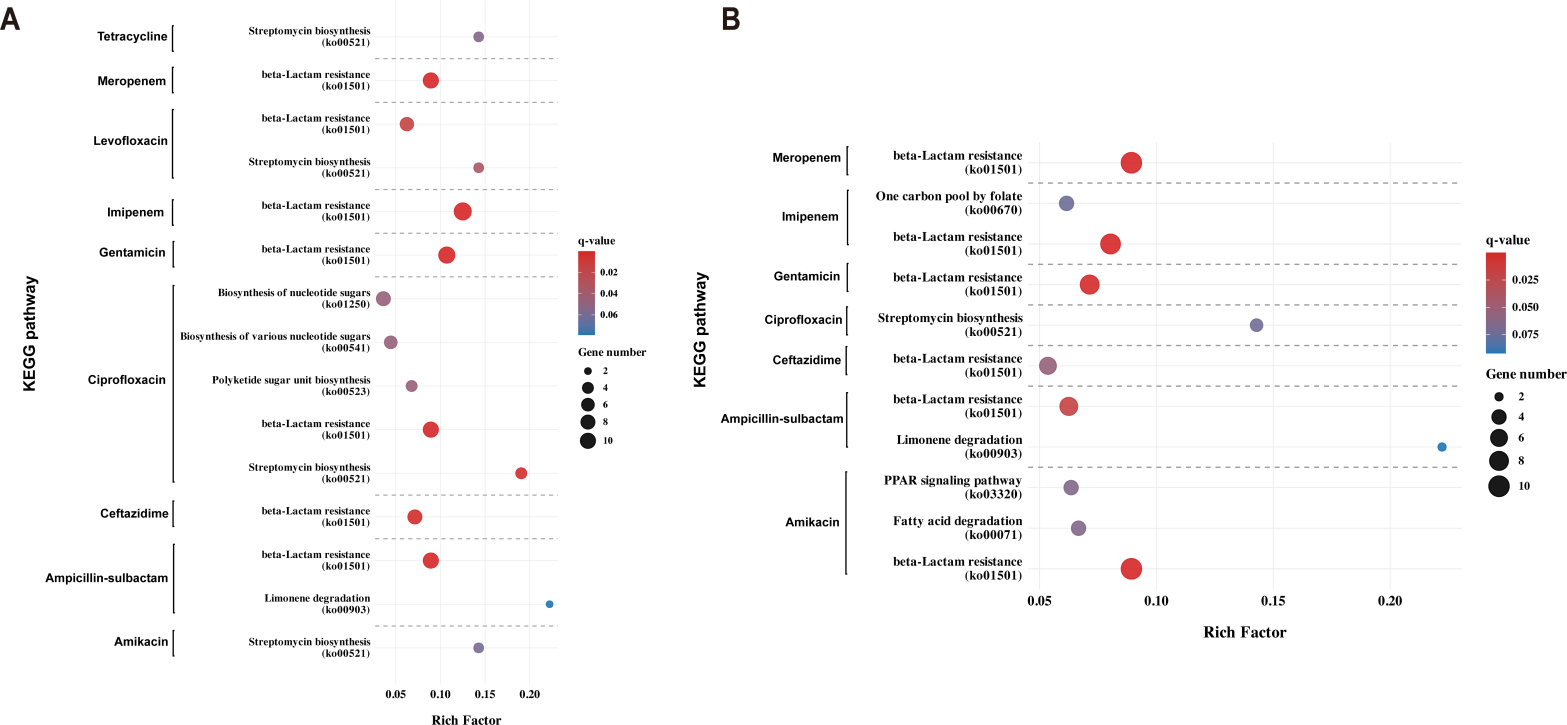
KEGG enrichment analysis of antibiotic resistance-associated KOs identified by machine learning approaches. (**A**) KEGG pathway enrichment for top 100 KOs ranked by XGBoost feature importance. (**B**) KEGG pathway enrichment for top 100 KOs ranked by XGBoost SHAP values. Significantly enriched pathways (q-value <0.1) are shown, grouped by antibiotic. Rich factor (*x*-axis) represents the ratio of genes in each pathway to the total number of genes in that pathway. Point size indicates gene number.

KEGG pathway enrichment analysis of the top 100 KOs from XGBoost and SHAP analyses revealed consistent patterns across multiple antimicrobial agents (Fig. 6A and B). Most notably, the β-lactam resistance pathways (ko01501) were significantly enriched (q-value <0.1) within the β-lactam and carbapenem groups. Previous studies have directly linked this pathway to resistance against the tested agents: ceftazidime resistance is predominantly mediated by extended-spectrum β-lactamases and AmpC enzymes within this pathway that specifically hydrolyze oxyimino-cephalosporin substrates (43), while ampicillin-sulbactam functions as a β-lactam/β-lactamase inhibitor combination that directly targets β-lactamase-mediated resistance mechanisms (44). In the XGBoost built-in importance analysis (Fig. 6A), streptomycin biosynthesis (ko00521) emerged as another recurrent pathway, particularly for tetracycline, levofloxacin, and ciprofloxacin. This enrichment is driven by the presence of streptomycin resistance genes (e.g., *strA* and *strB*), which are homologs of biosynthetic self-protection genes but are widely disseminated on broad-host-range plasmids alongside tetracycline and fluoroquinolone resistance determinants (45). This genetic linkage explains the pathway’s co-occurrence across diverse drug classes, highlighting the critical role of mobile genetic elements in facilitating multidrug resistance phenotypes (46).

### KEGG orthologs for multidrug resistance

Building upon the identification of individual resistance determinants, we next explored how these molecular mechanisms interconnect to drive MDR phenotypes. Given that SHAP values provide a more stable and consistent measure of feature contribution, we employed the top 10 SHAP-derived features for the following multidrug resistance analysis.

**Fig 7:**
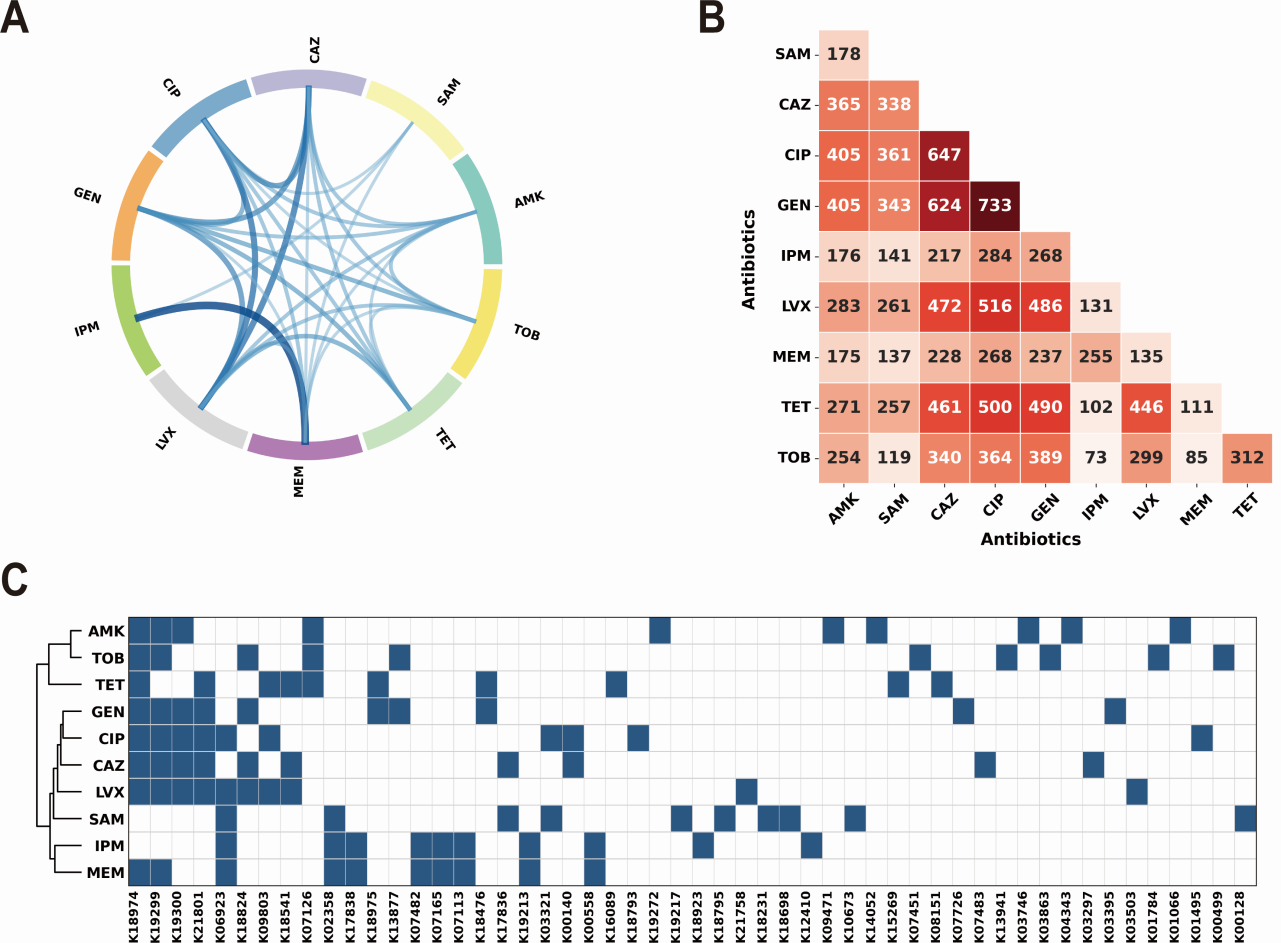
Multidrug resistance analysis revealing shared genetic architectures. (**A**) Chord diagram visualizing shared resistance determinants across antimicrobial classes based on top 10 KO terms for each agent. Chord thickness reflects the number of shared resistance-associated orthologs. (**B**) Clinical co-resistance matrix showing the number of isolates exhibiting co-resistance patterns between antibiotic pairs. The heatmap demonstrates substantial concordance with molecular connectivity patterns. (**C**) Hierarchical clustering heatmap of top 10 KEGG orthologs associated with resistance to each antimicrobial agent.

Leveraging the top 10 most important KO terms identified for each antimicrobial agent, we generated a chord diagram that visualizes the shared resistance determinants across antimicrobial classes (Fig. 7A). In this chord diagram, each antibiotic is positioned around the circumference, with connecting chords indicating shared KO terms between drug pairs—chord thickness reflects the number of shared resistance-associated orthologs. As expected, the thickest chord connects meropenem and imipenem, reflecting their shared carbapenem class and overlapping resistance mechanisms. Beyond this intra-class linkage, the diagram reveals extensive cross-class connectivity. Notably, ciprofloxacin and gentamicin emerge as central network hubs, extending chords to multiple distinct antimicrobial classes, which suggests that their resistance determinants frequently co-occur with those of unrelated agents.

To validate these computationally derived molecular networks against clinical phenotypes, we examined the co-occurrence of resistance phenotypes within our training set (Fig. 7B). The clinical co-resistance patterns demonstrate substantial concordance with the molecular connectivity observed in the chord diagram. Ciprofloxacin and gentamicin, identified as connectivity hubs in the SHAP-based molecular analysis (Fig. 7A), correspondingly exhibit the highest frequencies of clinical co-resistance (Fig. 7B): gentamicin shows extensive overlap with ciprofloxacin (733 isolates) and ceftazidime (624 isolates). Similarly, ciprofloxacin demonstrates broad co-resistance profiles, including substantial overlaps with ceftazidime (647 isolates) and levofloxacin (516 isolates). This convergence between molecular and clinical evidence supports the hypothesis that shared resistance genetic architectures facilitate the emergence of predictable multidrug resistance patterns in clinical isolates, providing a framework for understanding resistance co-evolution and informing surveillance strategies.

To identify common resistance mechanisms across antimicrobials, we generated a hierarchical clustering heatmap of the top 10 KEGG orthologs associated with resistance to each of the 10 antimicrobial agents (Fig. 7C). The resulting dendrogram identifies three primary clusters: a dedicated carbapenem cluster (imipenem and meropenem), an aminoglycoside-tetracycline cluster (amikacin, tobramycin, tetracycline), and a broad-spectrum cluster encompassing fluoroquinolones, cephalosporins, and gentamicin. This modular organization indicates that resistance evolution in *A. baumannii* follows predictable molecular pathways, where specific resistance determinants function as “hub” orthologs that simultaneously confer cross-resistance across related antimicrobial classes.

Notably, the most prevalent KO across multiple drug resistance profiles was K18974 (*sul1*), which encodes an altered dihydropteroate synthase. While primarily associated with sulfonamide resistance, its ubiquity as a top predictor across diverse drug classes reflects its role as a genetic marker for class 1 integrons—mobile genetic elements that frequently capture and co-express multidrug resistance gene cassettes (47). Following this, the aminoglycoside phosphotransferases K19299 [*aph(3*′*)-III*] and K19300 [*aph(3*′*)-II*] emerged as the next most frequent determinants. Both genes encode enzymes that inactivate aminoglycosides via phosphorylation, underscoring the centrality of enzymatic modification in driving high-level resistance phenotypes (48). Sharing the third rank with *aph(3*′*)-II*, K21801 (*iaaH*) represents a non-conventional resistance mechanism, encoding indole-3-acetamide hydrolase. This enzyme catalyzes the conversion of indole-3-acetamide to indole-3-acetic acid within the tryptophan metabolic network (49, 50). The widespread presence of *iaaH* in resistant isolates likely reflects its indirect but crucial role in regulating resistance through indole-mediated signaling, which has been demonstrated to activate efflux pumps, induce oxidative stress responses, and promote biofilm and persister cell formation (51).

Collectively, our findings demonstrate that antimicrobial resistance in *A. baumannii* follows predictable molecular trajectories, where shared genetic determinants facilitate multidrug resistance development along conserved pathways. The unexpected prominence of indole metabolism as a resistance hub illustrates how metabolic signaling networks intersect with canonical resistance mechanisms to amplify cross-resistance phenotypes.

## DISCUSSION

This study demonstrated that functional genomic representations through KEGG orthology annotations substantially enhance antimicrobial resistance prediction compared to traditional gene presence-absence approaches. The consistent 0.19%–1.02% improvement across all machine learning algorithms suggests that KO annotations capture critical biological features—including functional redundancy and metabolic pathway interactions. Our functional approach complements previous genomic studies in *A. baumannii* (52), which predominantly relied on SNPs or curated gene catalogs. While such methods are effective for known determinants, our findings confirm they often fail to capture resistance from novel functional elements, highlighting the need for pathway-centric models. Regarding data representation, utilizing raw, unnormalized KO counts preserved the sparsity of the feature space and prevented the noise amplification that global scaling often introduces to rare genomic elements. This data fidelity likely contributed to the superior and robust performance of algorithms adept at handling sparse, unscaled tabular data—such as XGBoost and TabPFN—compared to distance-based methods like SVM that mandate artificial feature scaling (53). Our analysis revealed several important biological insights into resistance mechanisms. The identification of canonical resistance genes such as *blaOXA-12* (54) validates our computational approach, as these carbapenemases represent well-established mechanisms of β-lactam resistance in *A. baumannii* (55). Remarkably, the prominence of K21801 (*iaaH*) across multiple resistance profiles highlights the underappreciated role of indole-mediated signaling in antimicrobial resistance (56). This enzyme catalyzes indole-3-acetic acid production within tryptophan metabolic networks, and indole has been demonstrated to regulate bacterial antibiotic resistance through activation of efflux pumps and oxidative stress responses (57). Consequently, the high predictive weight of *iaaH* identifies it not as a canonical resistance effector that directly neutralizes antimicrobials, but rather as a key regulatory and contextual contributor. This finding reinforces the concept that resistance mechanisms extend beyond direct drug-target interactions to encompass broader metabolic signaling networks. Furthermore, the enrichment of branched-chain amino acid biosynthesis pathways across multiple antimicrobial agents provides mechanistic insights into how metabolic flexibility contributes to resistance phenotypes (45). These pathways are essential for maintaining membrane integrity and cellular energy homeostasis under antibiotic stress (58), suggesting that metabolic vulnerabilities could serve as complementary therapeutic targets. The multidrug resistance analysis revealed that shared genetic architectures create predictable cross-resistance patterns, with ciprofloxacin and gentamicin emerging as hubs in this case. This observation has direct clinical implications, as resistance to these agents may serve as early indicators of broader multidrug resistance development (59). In addition, the strong concordance between molecular connectivity patterns and clinical co-resistance phenotypes validates the biological relevance of our computational predictions. This convergence between genomic architecture and clinical observations suggests that resistance evolution follows constrained pathways dictated by underlying genetic networks (60, 61), providing a framework for understanding and potentially predicting resistance co-evolution.

Nevertheless, the transition from *in silico* validation to clinical integration remains subject to specific operational and methodological constraints. Operationally, implementation is currently constrained by WGS turnaround time rather than inference speed (62), positioning our framework to capitalize on future rapid sequencing. Regarding cost, technological progress continues to lower WGS barriers, while optimized interventions yield offsetting healthcare savings (63). Furthermore, unlike deep learning approaches, our lightweight model requires no specialized hardware, thereby facilitating seamless integration into existing infrastructures (64).

Methodologically, several limitations warrant consideration. First, our reliance on KEGG orthology was a deliberate choice to leverage its strong pathway-centric annotations. While highly effective, this “macro-functional” view is not exhaustive. Future iterations could achieve a more comprehensive profile by integrating alternative classifications, such as Gene Ontology (65) or Clusters of Orthologous Groups (66). Moreover, the current approach omits sequence-level nuances within raw DNA and unannotated “hypothetical proteins.” To address this, large language models could be leveraged to generate feature embeddings directly from DNA sequences, capturing detailed information that pathway-based models miss (67). Finally, this study focused on *A. baumannii* to establish a robust, pathogen-specific baseline. While this successfully validates the feasibility of KO annotations, the current model remains species-specific. The next logical step is to expand this framework to other priority pathogens to create a generalized predictive tool.

Despite these limitations, the interpretability provided by our framework addresses a critical gap in current machine learning applications to antimicrobial resistance, where “black box” predictions have limited clinical acceptance (68). By identifying specific functional determinants, our method enables hypothesis-driven investigation of novel resistance mechanisms and facilitates the development of targeted therapeutic strategies. In summary, this study proposes and computationally validates a comprehensive machine learning framework that leverages functional genomic annotations to predict antimicrobial resistance phenotypes with enhanced accuracy and biological interpretability. The identification of metabolic signaling networks as resistance determinants suggests potential new avenues for therapeutic investigation. While these *in silico* findings are promising, they represent a crucial first step. Further investigations are necessary to expand this framework to additional bacterial pathogens and, most critically, to validate its clinical utility through prospective validation.

## Data Availability

All NCBI accession numbers for the genomes utilized in both the training and independent test sets are explicitly listed in Tables S1 and S2, respectively.

## SUPPLEMENTAL MATERIAL

Fig. S1 spectrum.02592-25-s0001.tif Phylogenetic tree.

Legend spectrum.02592-25-s0002.docx Legend for Fig. S1.

Supplemental tables spectrum.02592-25-s0003.xlsx Tables S1 to S7.

ASM does not own the copyrights to Supplemental Material that may be linked to, or accessed through, an article. The authors have granted ASM a non-exclusive, world-wide license to publish the Supplemental Material files. Please contact the corresponding author directly for reuse.
