# Supplementary material for: KEGG orthology-based machine learning reveals functional determinants of antimicrobial resistance in Acinetobacter baumannii
Source: Microbiol Spectr. 2026 May 7;14(6):e02592-25. doi: 10.1128/spectrum.02592-25 (PMC13228011; doi:10.1128/spectrum.02592-25)
Supplement: Legend — for Fig. S1. [file spectrum.02592-25-s0002.docx]

**Figure S1.** Phylogenetic tree rooted with reference strain Acinetobacter baumannii ATCC 19606 (red line) and annotated with top 15 sequence types. Color-coded outer ring represents ST distribution: ST-2 (red, n=347), ST-3 (blue, n=148), ST-32 (green, n=110), ST-1 (orange, n=48), ST-25 (purple, n=37), ST-10 (pink, n=27), ST-19 (cyan, n=22), Unknown (gray, n=19), ST-93 (yellow, n=14), ST-81 (dark red, n=13), ST-63 (blue-violet, n=12), ST-94 (forest green, n=11), ST-48 (hot pink, n=10), ST-150 (royal blue, n=10), ST-85 (lime green, n=9), and Others (light gray).
